# Roles of estrogens, estrogen-like compounds, and endocrine disruptors in adipocytes

**DOI:** 10.3389/fendo.2022.921504

**Published:** 2022-09-21

**Authors:** Fernando Lizcano

**Affiliations:** Center of Biomedical Investigation, Universidad de La Sabana (CIBUS), School of Medicine and Doctoral Program in Biosciences, Universidad de La Sabana, Chia, Colombia

**Keywords:** estrogens, menopause, estrogen receptor, endocrine disruptors, adipocytes

## Abstract

Women are subject to constitutional changes after menopause, which increases conditions and diseases prone to cardiovascular risks such as obesity and diabetes mellitus. Both estrogens and androgens influence the individual’s metabolic mechanism, which controls the fat distribution and the hypothalamic organization of the regulatory centers of hunger and satiety. While androgens tend to accumulate fat in the splanchnic and the visceral region with an increase in cardiovascular risk, estrogens generate more subcutaneous and extremity distribution of adipose tissue. The absence of estrogen during menopause seems to be the main factor that gives rise to the greater predisposition of women to suffer cardiovascular alterations. However, the mechanisms by which estrogens regulate the energy condition of people are not recognized. Estrogens have several mechanisms of action, which mainly include the modification of specific receptors that belong to the steroid receptor superfamily. The alpha estrogen receptors (ERα) and the beta receptors (ERβ) have a fundamental role in the metabolic control of the individual, with a very characteristic corporal distribution that exerts an influence on the metabolism of lipids and glucose. Despite the significant amount of knowledge in this field, many of the regulatory mechanisms exerted by estrogens and ER continue to be clarified. This review will discuss the role of estrogens and their receptors on the central regulation of caloric expenditure and the influence they exert on the differentiation and function of adipocytes. Furthermore, chemical substances with a hormonal activity that cause endocrine disruption with affectation on estrogen receptors will be considered. Finally, the different medical therapies for the vasomotor manifestations of menopause and their role in reducing obesity, diabetes, and cardiovascular risk will be analyzed.

## Introduction

During menopause, women experience an increase in fat mass due to multiple factors, mainly estrogen deficiency. Reduction in estrogen is associated with an increase in fat mass, especially in the visceral region, compared to the typical distribution of fat in women in a fertile state. However, the increase in weight observed after estrogen deficiency during menopause can be attributed in part to greater food consumption. This single factor does not entirely explain the increase in the accumulation of intra-abdominal fat because the deficit of estrogens can also act on the stimulation of physical activity and on the amount of accumulated lean mass (1). The distribution of fat in fertile women is the characteristic gynoid and fat is localized in the hips, thighs, and buttocks.

Similarly, estrogens can control both energy expenditure through modulation of the activity of the hunger and satiety centers, as well as thermogenesis at the level of the central nervous system. In fact, the reduction of estrogen receptor alpha (ERα) can increase body weight due to a reduction in energy expenditure and mild hyperphagia (2).

Despite a large amount of information published, the results of studies are not conclusive due to the complex mechanism of estrogen activity. One of the aspects that have aroused the most interest is the influence of environmental chemicals, some with recognized estrogenic effects, on the metabolism of adipose cells (3). Many of these products have a direct effect on nuclear receptors, including estrogen receptors, leading to a propensity for obesity. This creates a dilemma about the role of estrogens in metabolic regulation. While during menopause, estrogen deficiency can be a cause of obesity, some environmental chemicals with estrogenic effects have been blamed as triggers of obesity. In this review, we will evaluate the estrogen activity in the accumulation of energy, emphasizing its influence on the function of adipose cells.

## Mechanism of action of estrogens

Estrogens are hormones derived from cholesterol produced mainly in the granulosa cells of the ovaries. This hormone has a fundamental role in the development of female sexual organs and in the maturation of germ cells, whose purpose is to prepare for fertilization (4). Estrogens, like testosterone in men, have the peculiarity of redistributing the accumulation of fat mass within the body in certain places (5). In women of childbearing age, the accumulation of fat mass is mainly subcutaneous in the buttocks, legs, and hips (6). In men, the distribution is largely in the abdominal region, the visceral area, and the back. The main activity of estrogens is mediated by receptors belonging to the superfamily of nuclear receptors that act as transcription factors and mediate the gene expression effects of estrogens (7, 8). In these circumstances, once estrogens cross the plasma membrane, given their steroid structure, they bind to their receptors in the cytoplasm and then translocated to the nucleus, where bind to DNA and regulate gene expression.

Estrogen receptors have two main isoforms, called ERα and ERβ, with a wide distribution throughout the organism (9). These receptors can form dimers and bind specific areas of the promoter region of many genes in the so-called estrogen receptor response elements. Gene expression regulation by estrogens can also be indirect and not necessarily mediated by their receptors. This function was initially postulated because almost one-third of the genes regulated by estrogens do not have specific estrogen response element sites (2, 10). Molecular and biochemical studies have shown that this action of estrogens and their receptors can be mediated by other transcription factors that are activated through protein-protein interactions (11).

**Figure 1:**
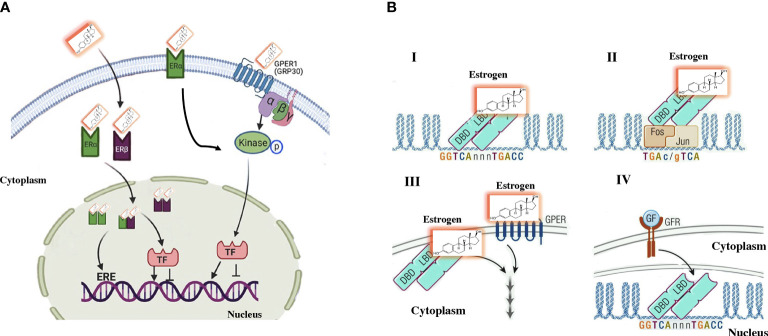
Traditional estrogen signaling mechanisms. **(A)** Genomic signaling: Estrogens cross the plasma membrane where they bind to estrogen receptors (ERs) in the cytoplasm. The estrogen–ER complex moves into the nucleus, where it forms homodimer and/or heterodimer complexes. These complexes bind to specific estrogen-sensitive elements (EREs) in DNA or recruit transcription factors. Nongenomic signaling: Estrogens can perform a non-genomic effect by binding to their own receptors located on the plasma membrane. Additionally, some ERα is in the plasma membrane that induce the signaling cascade. **(B)** A more direct characterization of the effects of estrogen signaling observed in **(A)** I. The genomic activity of estrogens includes the binding of ERs to EREs in the regulation area of genes. II. Mechanism that involves an indirect activity of estrogens include an indirect activity on gene regulation by binding to other transcription factors, such as the AP-1 DNA sequence that binds to dimers of FOS/JUN. Therefore, ERα is “bound” to DNA by the binding of FOS/JUN to its DNA motif AP-1. III. Non-genomic information is established by extracellular signaling that stimulates second messengers in the cytoplasm, there is not direct interreference on gene activity. Responses are mediated by specific G-protein coupled receptors or estrogen receptors on the membrane. IV. ER can be subject to ligand-independent activation by growth factor-mediated signaling that triggers the activation of intracellular signaling via the cell membrane receptor (GFR), which activates signaling pathways such as MAPK. The signal is mediated by estrogen receptors, which modulate the activity of specific genes. Modified (15).

Many of the activities of estrogens do not correspond to genomic activation, and some of the actions of estrogens have a faster effect than expected after genomic activation. Following the discovery of the G protein-coupled estrogen receptor (GPER1) (12) in the first decade of this century, it was observed that the influence of estrogens on the cardiovascular system and metabolism can be partly mediated by GPER1 (13). The binding of estrogens with GPER1 leads to signaling through adenyl cyclase-cAMP-protein kinase A and other second messenger cascades. The mRNA and protein expression of GPER1 has been observed in the blood vessels and the heart of several species (14). In other cells, such as adipocytes, liver cells, and muscle cells, the expression of the GPER1 protein is variable (**Figure 1**). Somehow, there is still controversy about the true role of GPER1 in the mediation of estrogen activity *in vivo* (16). Ligands-independent actions are ER-mediated effects seen after activating other pathways, such as IGF-1 receptor. This ligand-independent activation has been observed in the uterus, mediated by transcription factors that translocate ERα into chromatin (15, 17).

## Estrogens and estrogen receptors in adipose cells

Estrogens can also be produced in adipose tissue from androgenic precursors. Within adipocytes, estrogens are synthesized from androgens by aromatases, and their production increases according to the volume of adipose tissue (18). Estrone is the main estrogen produced in adipose tissue, derived from the aromatization of androstenedione, and acts as the main estrogen during menopause (19). The 17β-hydroxysteroid-dehydrogenase enzyme can convert estrone to estradiol in various tissues including primarily adipose tissue. The main estrogen receptor present in adipose tissue is ERα (20, 21). In humans, both ERα and ERβ exist in subcutaneous (SAT) and visceral (VAT) adipose tissue (22). However, in postmenopausal women, ERβ shows increased expression compared to premenopausal women in VAT (23). ERα expression has several functions in metabolism, especially affecting body fat accumulation glucose homeostasis, and energy expenditure (24).

Although the main estrogen receptor in adipose cells is ERα. ERβKO mice increase fat accumulation at inguinal levels, supporting the fact that these receptors also play a role in the control of fat distribution. Additionally, it has been observed that ERβ has an antilipogenic effect on adipose cells. ERβ deficiency increases the accumulation of white adipose cells at the subcutaneous level and increases the transcriptional signal of PPARγ. These observations show a negative effect of ERβ on PPARγ activity (25). In postmenopausal women, ERβ levels are increased in subcutaneous adipose tissue, while there is no variation in ERα. The discrepancy in the expression of these receptors, in part, could determine an unstable ERα/ERβ relationship. It is possible that differences in the expression of ERs in adipose cells may explain the characteristics of the change in adipose distribution among pre-postmenopausal women.

**Figure 2:**
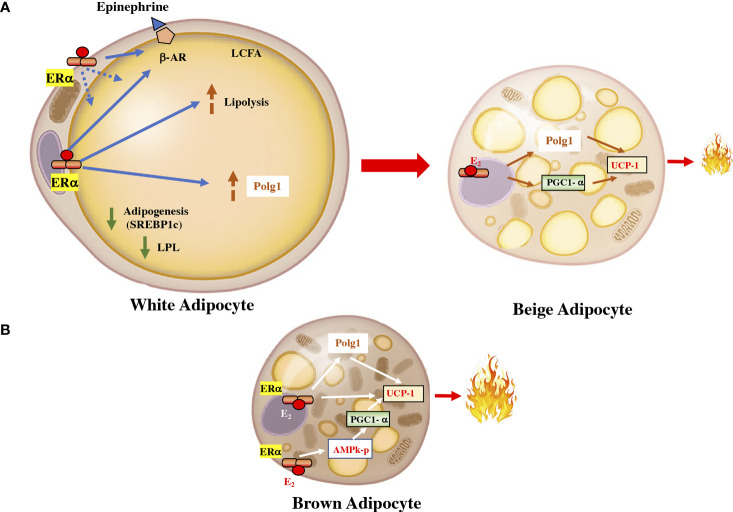
Estrogen in the fat cell. **(A)** In white adipocytes, the activation of the ERα receptor by estrogen downregulates lipoprotein lipase and increases the activity of the β-adrenergic receptor. It also increases the activity of Polg1 and increases mitochondrial activity. These effects can upsurge the transdifferentiation of white adipocytes into beige adipocytes, which are more energetically active. **(B)** In brown adipocyte cells, ERα can increase the expression of UCP1 by increasing the PGC1α coactivator through AMPK and by a direct effect on the receptor coactivator. UCP1, uncoupling protein 1; PGC1α, peroxisome proliferator-activated receptor gamma coactivator 1 alpha; ERα, estrogen receptor alpha; AMPK, AMP-activated protein kinase. LPL, lipoprotein lipase; β-AR, beta-adrenergic receptor; Polg1, mitochondrial DNA polymerase gamma; SREBP1c, sterol regulatory element-binding transcription factor 1c.

Estrogens regulates lipid homeostasis through the regulation of transcription factors belonging to the SREBP (sterol regulatory element-binding protein). This family is made up of three isoforms that are involved in specific processes: SREBP1c, which is involved in fatty acid synthesis, in adipose tissue homeostasis, and in insulin-induced glucose metabolism, mainly in the liver (26) (**Figure 2**). As in white adipocytes, in brown adipocytes, the ERα is predominant (27). However, the significance of ERβ expression has peculiar characteristics, given that the administration of a specific ERβ agonist induces SAT browning by increasing UCP1 expression. Selective agonists of ERβ, have shown anti-obesogenic effects, antidiabetic actions, precluded hepatic lipid accumulation, and reduced lipogenic gene expression levels (28).

The biology of adipose tissue has several physiological aspects regulated differentially between women and men. Some of these differences may be triggers for the greater predisposition to metabolic diseases in men. Studies in animals have observed that a high-fat diet increased the deposition of gonadal adipocytes, with hypertrophy in males and hyperplasia and hypertrophy in female mice (29). Notably, estrogen administration after ovariectomy protects females from hypertrophic changes and reduces inflammation and oxidative stress (30). Other observations have shown in mice that gonadal white adipose tissue in females is more metabolically active than in males, observing an improvement in lipolysis and the recruitment of brown adipocytes (31). There is growing evidence of the differential role of mitochondria in cellular pathologies between the sexes (32). Some observations have shown that the genes that regulate the mitochondrial activity, such as uncoupling protein 1 (UCP1), are subject to variation in the activity of estrogens. It is likely that mitochondrial function in adipose tissue is an important factor underlying sex differences (**Figure 2**). (33). A study conducted on humans observed that the energy expenditure of adipose tissue at rest was significantly higher in women than in men (34). They studied the gene expression of subcutaneous adipose tissue and observed a higher expression of genes with mitochondrial activity including UCP1.

Thus, the ability of white adipocytes to be more flexible phenotypically and to turn into more energetically active adipocytes, such as beige adipocytes, is greater in premenopausal women than in postmenopausal women and men (35). In addition, variation in sex hormone homeostasis can influence energy balance and glucose metabolism. These observations reinforce the importance of a gender-specific approach in personalized medicine. (36). This approach must consider the possibilities of regulating the action of gonadal hormones at the adipose tissue level. In this setting, it was observed that the specific activation of ERα in adipose cells can stimulate mitochondrial activity, upregulating proteins such as UCP1 and dynamin-related protein 1 (Drp1). The expression of the *esr1* gene encoding ERα, is negatively associated with fat mass, and its expression is higher in women. The expression of ESR1 can be modulated by environmental factors, including temperature, exercise, and caloric consumption (6, 35).

Although white adipocytes are the body’s most significant energy store, the presence of more metabolically active adipocytes, such as brown adipocytes or beige adipocytes, has aroused particular interest due to their influence on energy expenditure and prevention of metabolic diseases (37). Evidence of these metabolically active adipocytes is highlighted in colder temperatures. During exposure to cold, the mitochondria induce a change in the fatty acid metabolism substrate that, together with the increase in UCP1, generates more heat. The activation of white adipocyte browning significantly improves carbohydrate metabolism by weakening insulin resistance (38). Interestingly, women, who have more beige adipocytes before menopause, have a significant reduction in these adipocytes after menopause. Additionally, treatment with estradiol can increase the browning of white adipocytes in postmenopausal women (39, 40). Excess androgen inhibits brown adipogenesis in women, attenuates thermogenesis activation, and reduces mitochondrial respiration. These data provide a plausible mechanism that may contribute to reduced postprandial thermogenesis and the tendency to obesity in women with PCOS.

The fact that generates challenges in the studies of ERs signaling on obesity in humans is the fact that ER expression is not static. The equilibrium and levels of ERs (ERα isoforms and ERβ) can be transformed with aging, disease, and prolonged estrogen deficiency, all of which can alter the response to estrogen (41, 42). For example, ERs have been reported to be lower in postmortem coronary arteries from postmenopausal women compared with premenopausal women and lower in atherosclerotic coronary arteries compared with normal coronary arteries regardless of the menopausal state.

Brown adipocytes (BAT) are metabolically more active than white adipose tissue, and in adults is localized in the cervical, supraclavicular, axillary, mediastinal, and paraspinal (43, 44). BAT can be stimulated in adults and could have a relevant role in the treatment of obesity (45, 46). Experimental studies have shown that estrogens can intensify the thermogenic activity of BAT by increasing the expression of UCP1 mRNA (47).

ERα is expressed in BAT and is located mainly in mitochondria, suggesting that the mitochondria of BAT could be the target of estrogens and suggesting a role for ERα in mitochondriogenesis (48). The downregulation of ERs in adipose cells induces an increase in lipid accumulation and thus a gain in body weight. The deletion of *Esr1* in adipose cells reduces cellular respiration and the rate of fatty acid oxidation, along with an increase in the size of adipocytes, in both males and females (49). Experiments in mice with *Esr1* deletion in white adipose cells and brown adipose cells demonstrated the importance of *Esr1* in mitochondrial metabolism. Several genes regulating mitochondrial function were downregulated, including *Polg1*, whose protein product is involved in mitochondrial biogenesis. These authors showed that ERα controls the expression of *Polg1* and that a reduction in ERα leads to impaired mitochondrial remodeling. Reduced ERα induces metabolic damage in rodents and humans, such as promoting obesity. In fact, the authors point out that the action of ERα in adipose tissue could be a therapeutic target for obesity and other metabolic disorders (49, 50).

## Control of estrogens in the regulating centers of appetite and satiety

Evolutionarily, more than 300 million years ago, ERs already had an ancestral homolog that probably acted as a sensor of substances that had estrogen-like effects (51). These receptors could capture signals from substances with this estrogenic character from the environment or could even capture information from phytoestrogen substances (52, 53). Initial molecular physiology studies in mice showed that microinjection of estradiol into various brain areas could change the feeding behavior of animals, improving body composition (54).

In the central nervous system, the primary estrogen receptor is ERα, the functional relevance of this receptor in energy control has been demonstrated in studies in which ERα mutation induces obesity (55, 56). Interestingly, the elimination of ERα in the brain also affects the regulation of negative feedback by estrogens, which results in a higher level of 17β-estradiol in the blood (55). However, elevated 17β-estradiol in the circulation fails to prevent obesity, suggesting that in the brain, ERα plays a predominant role in the regulation of energy balance (57).

The hypothalamus is the nervous system region that controls food consumption, energy expenditure, and body weight homeostasis. The signaling mechanism of estrogen receptors in hypothalamic neurons has not been fully elucidated. It is thought that in addition to the activity of ERα on gene expression, part of its action is mediated by membrane receptors (58). Estrogen activity includes effects on different hypothalamic nuclei with divergent functions. Both the ventromedial nucleus (VMN), the arcuate nucleus (ARC), the preoptic area, and the solitary nucleus of the hypothalamus may have a preponderant role in the control of energy balance. For example, in the VMN, a reduction in the activity of ERα exclusively in this hypothalamic nucleus decreases the beneficial effect on the energy balance induced by the increase in estradiol (57, 59).

The ARC participates in controlling feeding. In this nucleus, estrogens produce a rapid increase in pro-opiomelanocortin (POMC) neurons, which can modulate food consumption, energy expenditure, and reproduction. POMC neurons also can secrete melanocortin-stimulating hormone (MSH), which acts in the paraventricular and lateral nucleus of the hypothalamus by binding to the melanocortin 3 (MC3) and melanocortin 4 receptors (MC4) (60). Under this consideration, estrogens can suppress the activity of neuropeptide Y through a form of ERα located in the membrane. Experiments in mice have shown that estrogens from the diet are regulated in accordance with the energetic state and ERα in the membrane such as those estrogens that have transcriptional effects controlling food consumption (58). The anorectic effect mediated by estrogens in the ARC includes the activation of the mammalian target of rapamycin and 5′AMP-activated protein kinase (AMPK) signaling. These pathways act as cellular sensors of nutritional status and cellular metabolism (61).

Other cerebral nuclei that can control feeding, which is in the brainstem, are the nucleus of the solitary tract and the nucleus of the dorsal raphe. These two nuclei have elevated levels of ERα, and increased neuronal activities of the nucleus of the solitary tract are associated with estradiol-induced anorexia in female mice (55). Microinjection of estradiol improves the effect of anorectic hormones such as cholecystokinin (62, 63). Despite a large amount of information available, the roles of other areas in feeding have yet to be elucidated in depth. Areas that are associated with food reward behaviors, such as the nucleus accumbens and the lateral hypothalamus, have been found to express ERα. In fact, estrogens influence the metabolism of some monoamines, especially dopamine, in the nucleus accumbens (64). It has been shown that estrogen increases dopamine synthesis and decreases its degradation and reuptake, increasing ERs levels. The effect of estrogen on the dopaminergic system is mainly observed in the prefrontal cortex, a region with high amounts of estrogen compared to other cortical areas (65). Furthermore, through its effects on the prefrontal area and limbic regions (such as the nucleus accumbes), estrogen influences emotional and motivational behaviors (66).

The menopausal transition is a period that has great relevance in the circumstances of cardiovascular risk in women. During this period the progressive reduction of the production of E2 by the ovaries. On average, women gain 2 to 3kgs over the course of the menopausal transition, with high inter-individual variability. Although many of the anthropometric variations observed around perimenopause can be attributed to estrogen deficiency. The progressive increase in FSH can have a negative impact on body composition in women (67). The Study of Women’s Health Across the Nation (SWAN) provided very compelling evidence that an accelerated gain in fat mass and loss of fat-free (lean) mass were related to the menopause transition rather than aging (68). Some intervention studies have observed that estrogen reduction can increase the accumulation of abdominal fat with a reduction in energy expenditure (69, 70). However, there is much disparity in the results of the studies and it is possible that this is due to the different influences of racial factors, lifestyle, cultural background, and other epigenetic factors that may intervene in the course of the menopausal transition period (71).

## Estrogens and energy regulation

Several experiments in mice have shown that ERα positively affects energy activity by promoting energy expenditure and reducing intake (72). In addition to the POMC neurons of the arcuate nucleus, estrogens can influence VMN neurons, where there is a high concentration of ERα (73). In fact, studies conducted with electrical, pharmacological, and hormonal stimuli have demonstrated the relevance of the VMN in thermogenic activation (74).

**Figure 3:**
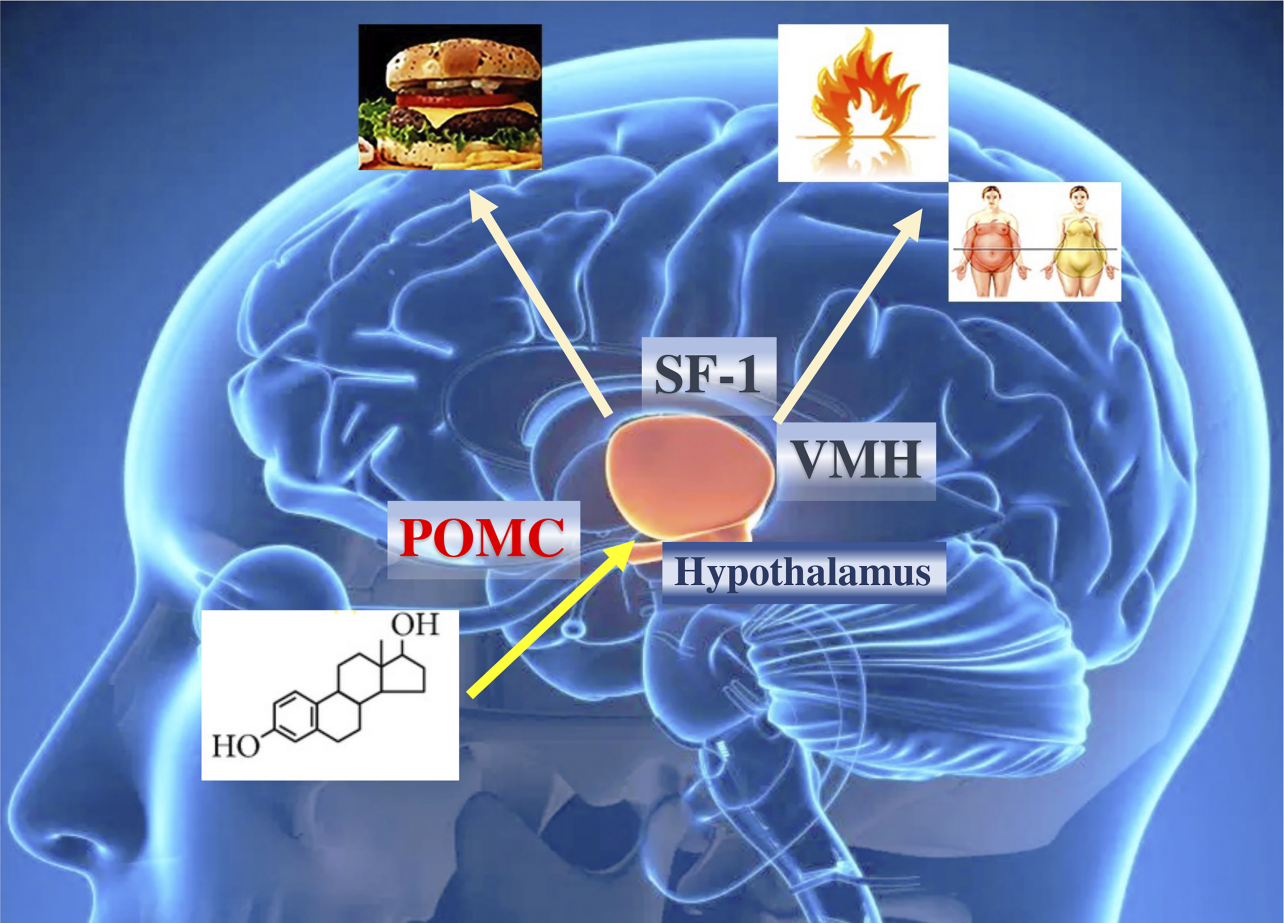
Effects of estrogens on hypothalamic and obesity control. ERα in the brain regulates body weight in both men and women. ERα in female SF1 neurons regulates energy expenditure and fat distribution. ERα in female POMC neurons regulates food intake. The effect of estrogens ultimately influences caloric expenditure, body composition, and food intake. POMC, pro-opiomelanocortin; SF1, steroidogenic factor 1.

The action of estrogens in the increase of thermogenic activity is mediated by neurons that express steroidogenic factor 1 (SF1), which induces a neuronal activation of the sympathetic system, generating an increase in UCP1, peroxisome proliferator-activated receptor gamma (PPARγ), PPARγ coactivator 1α (PGC1α) and the activation of β_3_-adrenergic receptors in adipose cells (75, 76). The simultaneous elimination of ERα in SF1 and POMC neurons generates a reduction in metabolism, hyperphagia, and severe obesity (55) (**Figure 3**). However, some of the effects of estrogens on the regulation of thermogenesis may be mediated by ERβ receptors. Selective ERβ agonists in female mice fed a high-fat diet upregulate the expression of UCP1 and reduce obesity. Likely, some of the effects of estradiol on brown adipose tissue (BAT) thermoregulation are mediated by ERβ (77).

Previous studies have shown that the influence of estrogens on metabolic control may be subject to the regulation of energy expenditure exerted by the neuronal nuclei of the hypothalamus. For example, inhibiting neurons of the VNM nucleus can establish an alteration in caloric expenditure without affecting physical activity (55).

The signaling pathway that has been described in the regulation of energy balance in the VMN nucleus is AMP-dependent kinase (AMPK). This molecule acts as an energetic sensor and develops its activity as a mediator of the action of estradiol. The microinjection of estradiol in the hypothalamus triggers an increase in sympathetic tone and body temperature with more significant activity of brown adipocytes. All these events can be inhibited with a block in the activity of AMPK, and more relevant, the activity of AMPK in the ARC nucleus is not necessary for the reduction in weight (56).

Current data have also described a new subset of ERα-positive neurons in the ventrolateral region of the hypothalamus that promotes female locomotor activity. These neurons express Tachinia (Tac), which is closely related to physical activity in women. The deterioration of the development of these neurons causes inactivity and obesity without changes in the thermogenesis of BAT (78, 79). In general, this evidence suggests that estradiol probably induces specific effects on energy homeostasis according to neuronal populations of the VMN. In the neurons of the VMN, the silencing of the neurons that express the gene that codes for repression (*Rprm)* in females increases the body temperature without a significant variation in physical activity (57). The RPRM protein exerts a cell cycle monitor function and has evident hormone-dependent expression in tissues such as the pituitary (80).

The functional dissection of sex differences in the neural circuits that control food intake and energy expenditure is essential to understanding the biological basis of sex differences in body weight control. Additionally, it can establish the circumstances that are related to energy control when there is a hormonal change as marked as in menopause. Menopause is associated with significant increases in visceral abdominal fat and body weight, without an increase in caloric intake. The decrease in total energy expenditure can contribute to these changes in fat accumulation in women entering menopause. Therefore, factors that contribute to decreased energy expenditure, such as variations in thermogenesis, could be primary risk factors for postmenopausal obesity. The molecular mechanism by which ERα signaling in the neurons of the VMN that are Tac1^+^ and Rprm^+^ drives changes in physical activity or thermogenesis will be of interest for the potential treatment of postmenopausal obesity. In adulthood, it is possible that estrogen signaling activates the Tac1^+^ and Rprm^+^ neuron groups in women to increase their energy expenditure. During menopause, the energy expenditure that accompanies the abrupt decrease in circulating sex hormones can affect the neurons that code for these proteins and thereby reduce energy expenditure (81, 82).

**Figure 4:**
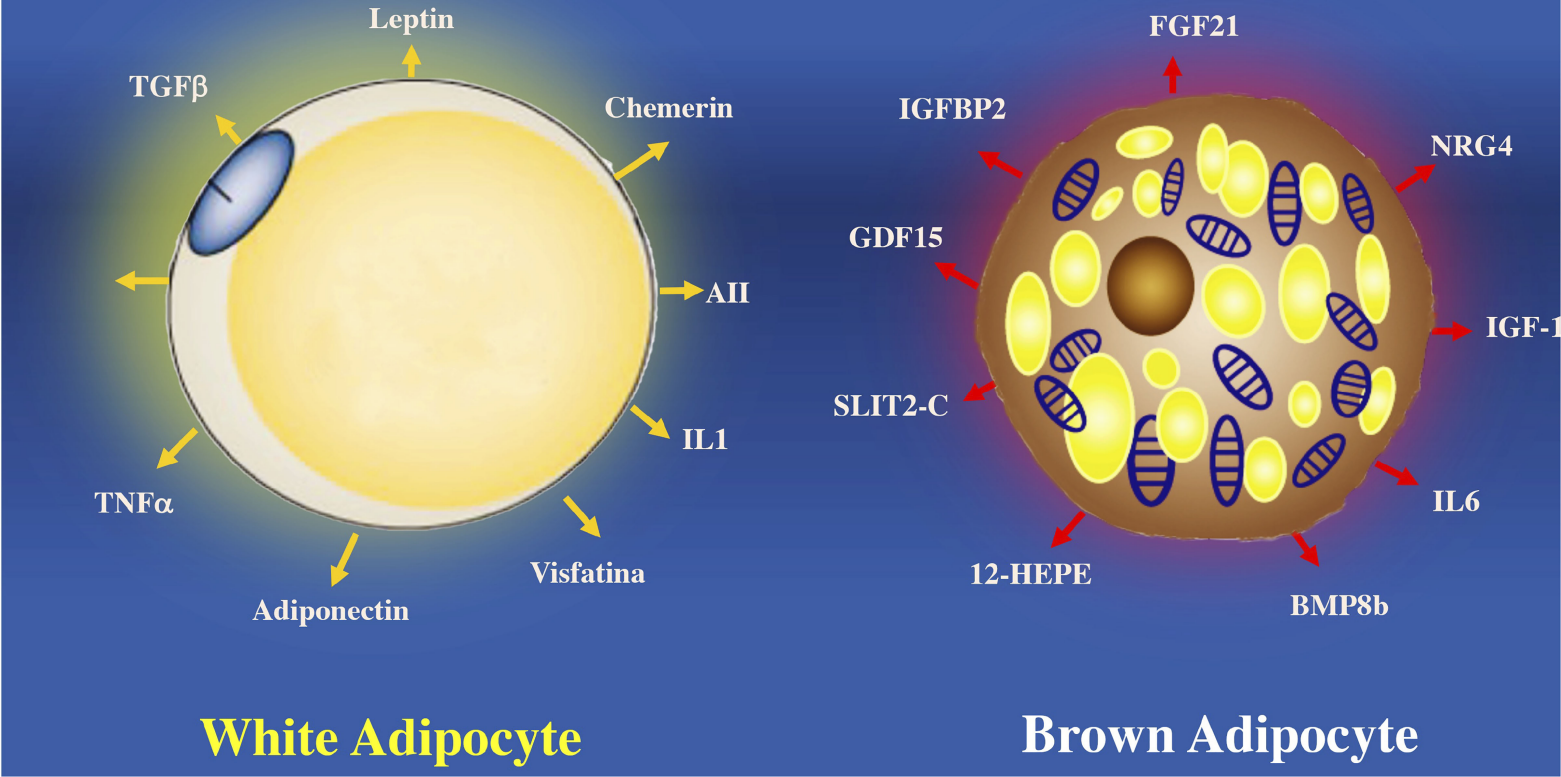
Estradiol availability affects the regulation of enzymes involved in glycolysis in the cytoplasm and in the tricarboxylic acid cycle (TCA) in mitochondria. Estrogens increase the activity of some glycolysis enzymes listed in the figure (hexokinase, phosphofructokinase, enolase, pyruvate kinase). E_2_ improves the glycolytic–pyruvate–acetyl-CoA pathway to generate the electrons necessary for oxidative phosphorylation and thus the generation of ATP to maintain the use of glucose as a primary source of fuel.

Enzymatic control of metabolic pathways that control energy production through glucose are regulated by estrogens. Enzymes like hexokinase, phosphoglucoisomerase, phosphofructokinase, aldolase, glyceraldehyde 3-phosphate dehydrogenase, phosphoglycerate kinase 6-phosphofruct 2-kinase, fructose 2,6-bisphosphatase, and the glucose transporters Glut3 and Glut4, increase after estrogen therapy (83, 84). Additionally, estrogens regulate the activity of Krebs/tricarboxylic acid cycle enzymes, such as citrate synthase, mitochondrial aconitase 2, isocitrate dehydrogenase, and succinate dehydrogenase (85) (**Figure 4**).

## Estrogen and adipokine secretion

In women of childbearing age, one of the best-characterized adipokines, leptin, is closely related to serum estrogen levels. Leptin is an adipokine with a known pro-inflammatory effect, which affects appetite, satiety, and energy expenditure. (86). Estrogens increase the sensitivity to leptin of energy controllers in the hypothalamus, and an increase in estrogens exerts a positive effect on the expression of leptin receptors in the ARC (87).

Leptin plays a major role in the central control of energy storage and negative feedback controls weight loss. Leptin levels are closely related to the amount of adipose tissue. When there is a significant loss of weight, leptin levels are reduced with a decrease in its levels in the hypothalamus, increasing the search for food. The intake increases the amount of stored energy, leading to a rise in free acids in adipose tissue, restoring leptin levels and reducing appetite (88). Although leptin is elevated in humans with obesity, these elevated levels do not influence reducing obesity. In part because obesity triggers a state of leptin resistance. In which inhibitory signals act on leptin receptor activity, increased stress on the endoplasmic reticulum, increased gliosis, and other inflammatory stimuli that induce leptin resistance. (89, 90).

Another adipokine that has an important systemic effect is adiponectin, which is the most abundantly expressed adipokine with an anti-inflammatory function, found in human serum in the μg/ml range (89, 91). Unlike all other adipokines, it is produced predominantly in the adipose tissue of the bone marrow (92). Adiponectin forms aggregates that circulate in the different forms of molecular weight. The high molecular weight form has the highest effect on improving insulin sensitivity (93). Adiponectin has two main receptors AdipoR1 and AdipoR2, which are expressed in vascular endothelial cells, adipocytes, monocytes, macrophages, and myocytes (94, 95). Adiponectin plays a protective role against cardiovascular diseases by inhibiting the formation of foam cells, the expression of adhesion molecules, and the interactions between endothelial cells and monocytes (91, 96). It also inhibits the synthesis of proinflammatory cytokines such as IL-6, IL-18, and TNF-α. Adiponectin induces adipogenesis through an expansion of adipose tissue with the formation of a fat pad that reduces inflammation, maintaining glucose homeostasis with a reduction in insulin resistance (97).

PPARγ agonists increase adiponectin synthesis (98). Adiponectin levels can be increased by factors such as glucocorticoid therapy, fasting, or conditions that increase the extension of adipose tissue in the bone marrow. In contrast, a reduction in adiponectin levels is observed after oxidative stress, smoking, and obesity itself (99, 100). Serum levels of adiponectin are higher in women than in men, in fact, plasma levels of adiponectin correlate with estrogen concentration. (101). Oophorectomy of adult mice reduces adiponectin, which is reversed with estrogen replacement (102, 103). Studies performed in MDA-MB-231 cells with ectopic ERα/ERβ expression manifest that the adiponectin-sensitizing action of E2 is executed through its classical nuclear receptors (104).

Resistin is an adipokine from white adipose tissue that plays an essential role in the appearance of complications of obesity. Elevated plasma Resistin levels increase inflammatory processes that activate insulin resistance and predict the forthcoming development of type 2 diabetes mellitus (105, 106). *In vitro* studies have shown increased proinflammatory cytokines such as TNF-α and IL-1. In addition, the functional modification of metabolic controller AMPK may be involved in resistin-mediated insulin resistance (107). Visfatin is an adipokine secreted by endothelial cells, macrophages, and adipocytes. Recently, an association between increased visfatin levels and increased risk of cardiovascular events has been observed in patients with diabetes mellitus type 2. (108, 109).

In contrast to white adipose tissue, BAT secretes substances called batokynes with multiple effects in different organs. Compared to white adipose tissue, BAT increases heat production because optimal oxygen consumption rapidly metabolizes fatty acids, favoring optimal oxygen consumption (110, 111). Many environmental or molecular stimuli induce BAT differentiation. The embryological development of BAT precedes white adipocytes due to its relevant role in thermogenic regulation in the newborn. BAT develops from a subpopulation of the dermomyotome that expresses molecular markers such as paired Box 7 (Pax7), engrailed-1 (En1), and myogenic factor 5 (Myf5) (112, 113). BAT can secrete cytokines that influence different tissues such as Follistatin a glycoprotein that reduce the activities of members of the transforming growth factor family and prevent diet-induced obesity (114).

**Figure 5:**
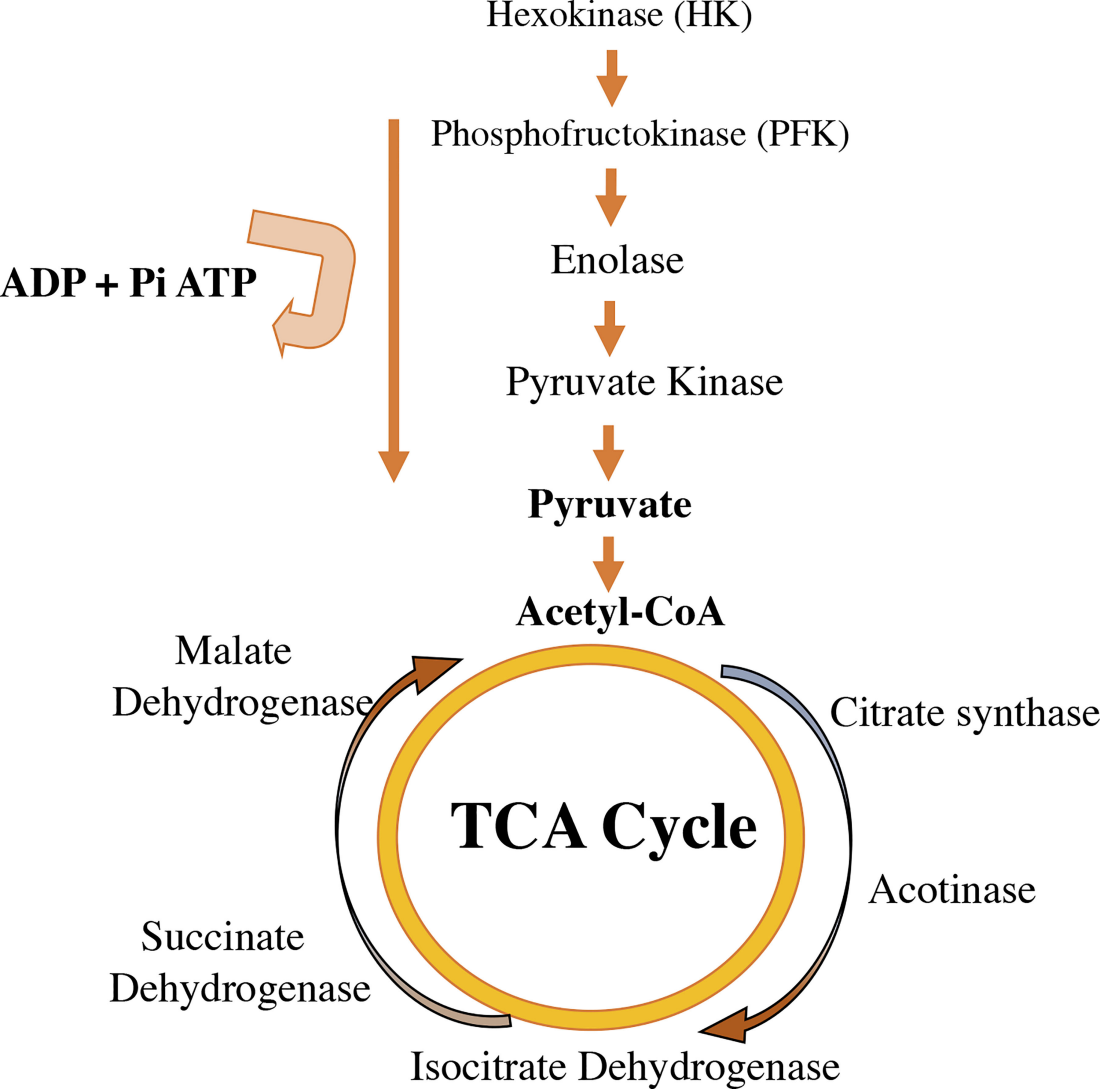
Proteins secreted by white adipocytes (adipokines) and brown adipocytes (“batokines”). The recognition of adipose cells as an endocrine organ is relatively recent. Many of the cardiovascular complications observed in obesity are a consequence of the altered secretion of these proteins in hypertrophied white adipose cells. Brown adipocytes secrete proteins that regulate some of the most important tissues involved in the control of body weight, lipid and carbohydrate metabolism. BMP8b, bone morphogenetic protein 8b; IGF-1, insulin-like growth factor 1; IGFBP2, insulin-like growth factor binding protein 2; IL6, interleukin 6; NRG-4, neuregulin 4; SLIT2-C, cleft 2 homologous protein; NRG4, neuregulin 4; ANGPLT2, angiopoietin-like peptide 2; IL1, interleukin 1; FGF21, fibroblast growth factor 21; TNFα, tumor necrosis factor alpha; 12-HEPE, 12-hydroxyeicosapentaenoic acid; AII, angiotensin II.

BAT secretes other proteins such as the C-terminal fragment of the cleft guide ligand 2 (SLIT-C). SLIT-C belongs to the family of secreted cleft proteins that play important roles in the chemotaxis of inflammatory cells, induce metabolic processes, and increase white adipocyte browning (115). Myostatin (GDF8) and growth differentiation factor 15 (GDF15) proteins members of the transforming growth factor family are involved in controlling neuronal circuits related to hunger. GDF15 develops insulin resistance and obesity by increasing the expression of the thermogenic genes (116). Another factor that regulates the physiology of adipocytes is Fibroblast growth factor 21 (FGF21). Which is secreted by BAT and protects against hyperglycemia and hyperlipidemia in mice (117). FGF21 analogs reduce dyslipidemia and hepatic steatosis in obese patients with type 2 diabetes mellitus, although they do not improve glucose or body weight control (118). While FGF21 seems to have anti-inflammatory effects on white adipocytes, it remains to clarify whether FGF21 has a similar action on BAT (118) (**Figure 5**).

## Estrogen-like compounds and endocrine disruption

Some substances found in plants or synthetic chemicals can affect different aspects of estrogen activation. Because of their multifaceted actions, they were grouped into critical characteristics beneficial to discerning their mechanisms of action (119). The undesirable symptoms observed in menopause can be reversed with chemical compounds with mild estrogenic effects. This is the case for tibolone, a synthetic substance with mild activity on estrogen, androgen, and progesterone receptors (120). Tibolone can improve vasomotor symptoms during menopause and can affect cardiovascular irrigation and body weight in postmenopausal women (121, 122). The treatment for one year with tibolone decreased fat mass, but tibolone combined with 17β-estradiol and norethindrone for two years did not significantly decrease fat mass (123). The treatment with hormone replacement therapy and tibolone improves the waist/hip ratio in menopausal women without observing a reduction in body weight. (123). Tibolone has shown some beneficial effects on cardiovascular risk in postmenopausal women (124).

Genistein is an isoflavone that has a structure like that of 17β-estradiol and can bind to ERα and ERβ to mimic the actions of estrogens in target organs. Due to its effect on ERs, genistein is frequently used by postmenopausal women to reduce vasomotor symptoms. Genistein administrated in higher doses increase the oxidation of fatty acids and reduce the accumulation of fat in the liver (125). In this way, genistein reverses the accumulation of trunk fat in postmenopausal women and ovariectomized rodent models.

**Figure 6:**
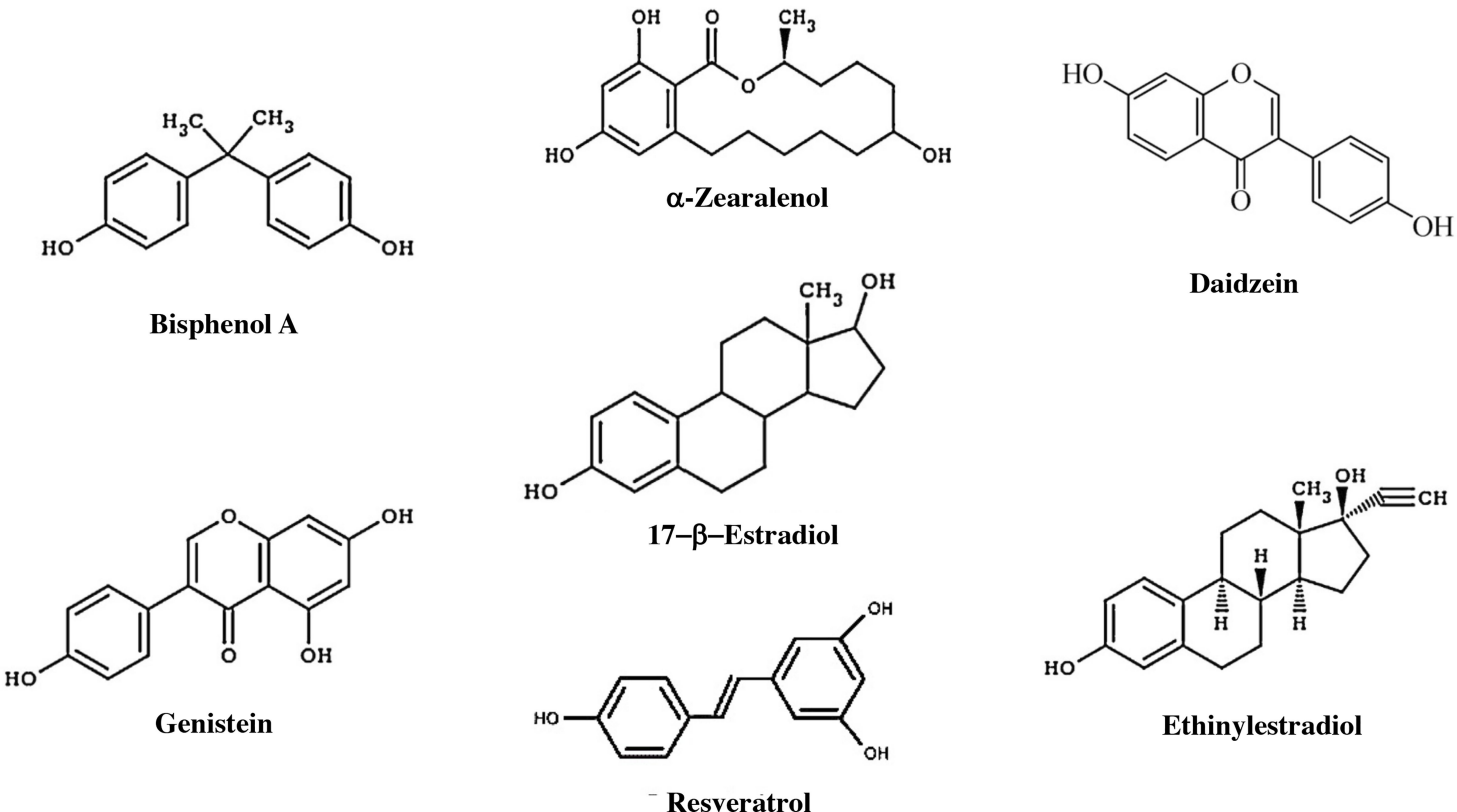
Components with estrogenic effects. Estrogens, some endocrine disruptors, and other natural products that have an estrogenic effect. Bisphenol A is an organic compound that is used to manufacture polycarbonate polymers and epoxy resins. α-Zearalanol is a natural compound observed in fungi that is a growth promoter used in veterinary medicine. Daidzein is a natural isoflavone compound found in products such as soy. Genistein is an isoflavone found in several plants, including soy. DDT, dichlorodiphenyltrichloroethane. Resveratrol is a compound found in grapes that has an estrogenic effect and may influence aging. Ethinylestradiol is a synthetic compound that is used as a contraceptive.

The endocrine system preserves homeostasis of the body and influences almost all cells and organs of the body by controlling metabolism, growth, and energy. Because detection techniques have improved considerably, it has been discovered that the endocrine system is susceptible to small changes in hormone concentrations. As a result, concern has grown about environmental pollutants that can mimic hormonal activity in recent years. Endocrine-disrupting chemicals (EDCs) are defined as chemicals that affect any aspect of hormonal action (126) There are approximately 85,000 chemical products available on the market, and of these, it has been shown that approximately 1000 have endocrine-disrupting properties (127, 128) (**Figure 6**).

Multiple chemical substances affect the activity of estrogen receptors, and their mechanisms of action are diverse. One substance that has an important role in endocrine disruption is bisphenol A (BPA). This chemical is useful for the manufacture of plastics for everyday use. Part of its phenolic structure resembles the A ring of estradiol, which allows it to activate ERs (129). Several experimental studies in animals and some in humans have shed new light on molecular mechanism of action of BPA (130). BPA binds ERα and ERβ, as well as GPER and membrane ER (131), the concentrations of ERα and ERβ can be modified by variations in the methylation of their promoters induced by BPA (132). The nongenomic effects of BPA in breast cancer cells have been associated with the activation of GPER1 (133).

Additionally, BPA has an epigenetic effect mediated by the enrichment of the trimethylation of histone three lysine 4 (H3K4) and the specific H3K4 methyltransferases, whose effect is the activation of the genes. BPA can induce the activity of the estrogen response element-binding region in the HOX antisense RNA promoter (HOTAIR). HOTAIR is a long noncoding RNA (lncRNA) that influences the appearance of tumors such as breast and prostate cancer (134, 135). BPA can alter the methylation patterns of specific genes and change their expression. Initial studies with agouti mice showed a change in the coat and weight of the mice after exposure to BPA, which was prevented by folic acid supplementation (136). BPA affects the activity of DNA methyltransferases by altering the expression of these proteins in the hypothalamus (137).

A family of organic compounds manufactured for use in different types of industrial applications is polychlorinated biphenyls (PCBs). PCBs are a family of chemical compounds with variations in the position of a chlorine group that gives them functional specificity. Although its use was prohibited for several years, it is general for industrial and commercial applications in some places. They are frequently found in electrical equipment, plastic, plasticizers in paint, and rubber products, as well as in dyes, pigments, and especially carbonless paper (138). One of the properties of PCBs, which have popularized their use, are their lipophilic properties and their resistance to decomposition. The persistence of PCBs in the environment passes through the soil and water and eventually accumulates in plants and crops. The adverse effects caused by PCBs include an increase in cell proliferation, affecting cell immunity, the nervous system, and an alteration in the function of endocrine glands (139). PCBs have demonstrated *in vitro* proliferative effects in breast cancer cells (140). In the brain, specifically at the paraventricular nucleus of the hypothalamus, exposure to PCBs reduces the number of cells expressing ERα in female rats (141).

## Estrogen therapy and obesity

Estrogen deficiency is the primary pathophysiological mechanism underlying the symptoms of menopause. Therapeutic options for the symptoms of menopause include hormonal preparations, nonhormonal drugs, and nonpharmacological therapies. Hormone replacement therapy (HRT) with estrogens is the most efficient therapy for vasomotor symptoms, sleep disorders, and emotional changes in postmenopausal women. Both experimental studies and clinical trials have shown the benefits of HRT on menopause symptoms, especially during the so-called “therapeutic window”. Around 10 years of menopause and under 60years of age, formulation of estrogen + progesterone has been shown effective. However, therapy with estrogens or the combination of estrogens and progesterone has also shown deleterious effects including the proliferation of endometrial cells, breast cancer, and vaginal bleeding (142). Among the therapeutic alternatives is tibolone, which has androgenic, progestogenic, and estrogenic effects. The estrogenic effects of tibolone are expressed mostly in the brain, vagina, and bone tissue, while they seem to be less important in the endometrium. In this regard, the risk of hormone-dependent cancers is lower than with some other HT protocols, as has been reported in recent years (143).

The position of the North American Menopause Society on HRT provides simple recommendations for the management of menopause. It suggests that HRT can help reduce abdominal fat accumulation and weight gain associated with transitioning to menopause (144).

HRT raises lean mass content and decreases abdominal fat content (145–147). The mechanism of this process is not fully understood. Studies in ovariectomized female mice treated with estrogens show increased lipid oxidation and more significant energy expenditure without affecting energy consumption. These results are consistent with observations of higher energy expenditure after the activation of ERα in VMN neurons in the hypothalamus (55, 148). Estrogens influence glucose homeostasis through increased glucose transport in cells. In contrast, a deficiency of estrogens has been associated with a progressive deterioration of insulin secretion after stimulation with glucose and increased insulin resistance. HRT furthermore contributes to the significant reduction in the diagnosis of type 2 diabetes mellitus in menopausal women. (149, 150). Despite these observations, the studies’ designs and contexts that show estrogens’ influence on glycemic control and insulin resistance are highly variable. The effect is better observed in women who have begun menopause more recently (151).

The progesterone supplement aims to protect the uterus against the consequences of systemic estrogen therapy, such as endometrial pathologies. The risk/benefit ratios of all treatment options should be considered, considering the nature and severity of the symptoms and the individual risks related to the treatment. (152). Estrogen/progestogen-based hormone replacement therapy in menopausal women decreases visceral adipose tissue and lowers fasting serum glucose. Additionally, estrogen/progesterone therapy can have a positive impact by reducing total cholesterol and relative low-density lipoprotein levels (153, 154). Therefore, diminished cardiovascular risk factors increase during menopause.

Using phytoestrogens as adjuvant therapy to reduce cardiovascular risk in menopausal women has also yielded contradictory results. In addition to individual clinical trials, a couple of meta-analyses evaluated the influence of dietary phytoestrogens on women’s body composition. In a study, a group of 272 menopausal women was supplemented with soy isoflavones. Isoflavone helped to reduce weight after 52 weeks of therapy. The beneficial influence on body weight of isoflavone was detected at low doses and during quiet periods of treatment, as well as in patients who were not obese (BMI<30m/kg2) at the start of therapy (155).

A recent meta-analysis showed a beneficial effect of phytoestrogen therapy on body composition in 1,880 postmenopausal women. A reduction in waist/hip ratio was observed in relation to a decrease in visceral fat. In contrast, no significant changes were observed in body weight, BMI, total fat mass, or percentage of body fat (156). Notwithstanding the divergence of the results of both studies, it can be considered that supplementation with phytoestrogens has a beneficial effect on visceral fat accumulation. Therefore, phytoestrogens not only have a favorable impact on the number and distribution of adipose cells but also affect the metabolism and production of adipokines. Phytoestrogens could effectively treat complications related to visceral obesity, at least in selected subgroups of patients (157).

## Conclusion

The vast amount of information about the activity of estrogens and their influence on different metabolic areas has significantly developed in recent years. However, hormone therapy for women during menopause is a great challenge deciding the risks and benefits. There is consensus about the benefits of hormone therapy in improving symptoms of estrogen deficiency in women under 60 years of age. HRT reduces the risk of coronary disease, cerebrovascular accidents, obesity, and diabetes mellitus 2. However, the decisions regarding the indication of hormone therapy require the evaluation of each patient and their potential risk, especially in the presence of breast cancer. New advances on the impact of ERs on the hypothalamic regulation of the control of energy expenditure and the role of ERs in the physiology of muscle and adipose cells have demonstrated their importance in the physiology of body composition. It will be necessary to standardize the research methods to determine the effects of HRT. It is also essential to highlight the role of new synthetic compounds with estrogenic effects in improving body weight and mitigating cardiovascular risk in postmenopausal women.

## Author contributions

The author confirms being the sole contributor of this work and has approved it for publication.

## Funding

This work is part of an investigation Funded by the Research Department from the Universidad de La Sabana (DIN) with the grant MED-287-2020.

## Acknowledgments

This work has been completed thanks to the collaboration of the members of the research center of the University of La Sabana. Especially Felipe Arroyave and Yomaira Uscategui.

## Conflict of interest

The author declares that the research was conducted in the absence of any commercial or financial relationships that could be construed as a potential conflict of interest.

## Publisher’s note

All claims expressed in this article are solely those of the authors and do not necessarily represent those of their affiliated organizations, or those of the publisher, the editors and the reviewers. Any product that may be evaluated in this article, or claim that may be made by its manufacturer, is not guaranteed or endorsed by the publisher.
